# Supplementary material for: Uncovering narrative aging: an underlying neural mechanism compensated through spatial constructional ability
Source: Commun Biol. 2025 Jan 21;8:104. doi: 10.1038/s42003-025-07501-5 (PMC11751312; doi:10.1038/s42003-025-07501-5)
Supplement: Supplementary file 2 — Supplementary Materials [file 42003_2025_7501_MOESM2_ESM.pdf]

# Supplementary Materials

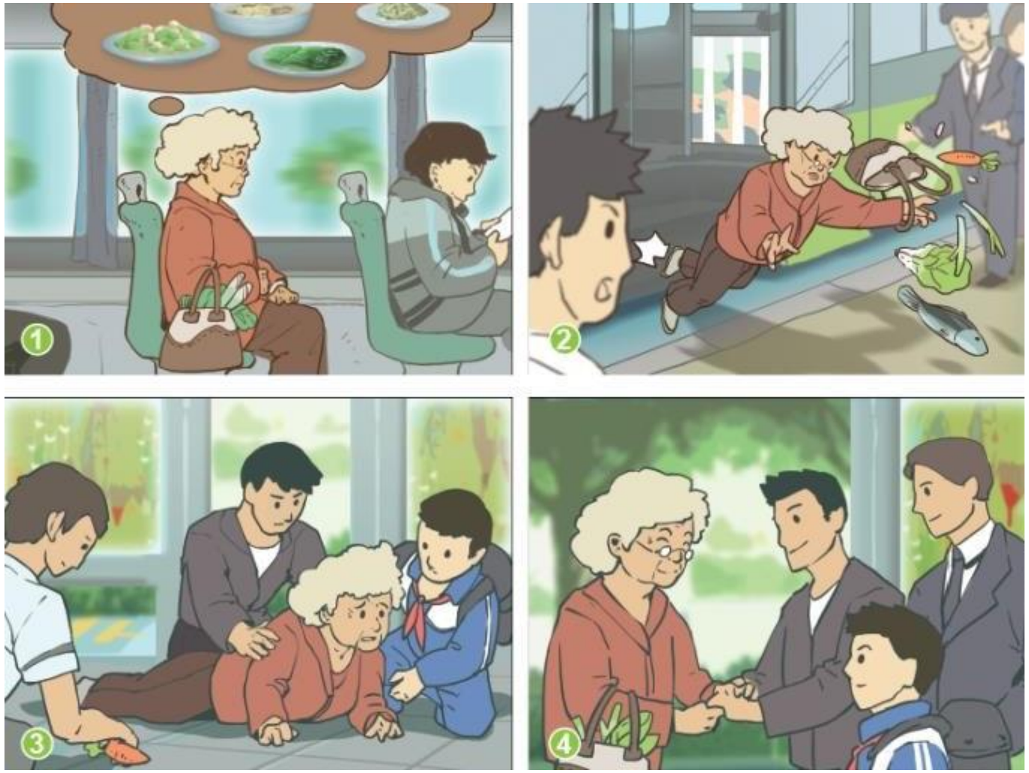

Supplementary Figure 1 Material for the narrative task

This figure was originally created by the authors for this publication and is protected by copyright. All rights reserved.

Supplementary Table 1 Macrostructure subscale: Story elements, definitions, and scoring criteria

| Macro elements | Scores | | | |
|---|---|---|---|---|
| | 0 | 1 | 2 | 3 |
| **Role**<br>The subject who performs an action | No protagonist | The story contains one main character, and the supporting characters are referred to using nonspecific quantities (e.g., "the old lady" or "Mrs. Zhang," while other characters are referred to more generally as "people around," "people nearby," and "kind-hearted people," etc.). | The story contains one main character and 1-2 supporting characters who are specified (e.g., grade school student, young man, father-son relationship, etc.). | The story contains one main character and three supporting characters who are addressed by appropriate names (such as grade school student, young man, middle-aged person). |
| **Background**<br>Location and time information | No time or location information | 1. Only one background information is provided. 2. There is no clear causal connection, such as "riding the bus" or "buying vegetables," with only one of these mentioned. | 1. Two pieces of background information are provided (taking bus and buying vegetables). 2. There is no reasonable and clear causal connection between background information. | 1. Specific times are given with specific names (such as early morning).<br>2. Two or more pieces of background information are provided.<br>3. There is reasonable and clear causal connection between background information. |
| **Initial event (IE)**<br>The event that triggers the role to act | No indication of an initial event. | The event is described, but it is not explained how "the event prompted the character to take action" (only mentioning falling down, not mentioning falling down when getting off the bus). | The event is described, with the action taken by the character was prompted by the event (falling down when getting off the bus, with a clear causal connection). | Two or more events are described, prompting different actions to occur (forming a complex and continuous story), such as falling down when getting off the bus and vegetables spilling everywhere. |

| | | | | |
|---|---|---|---|---|
| **Plan**<br>The roles express the idea of deciding to perform a certain action | No indication that the role plans to perform an action | Includes one statement of plan that is not related to the IEs. | Includes one statement of plan that is indirectly related to the IEs e.g., the old lady fell down because the bus was too slippery (this reason is not reflected in the pictures). | The character's plan to take action is outlined, but the action is not directly related to the IEs (it can be reasonably inferred from Picture 1 that the reason the old lady fell down was because she was distracted, thinking about what vegetables to cook for lunch). |
| **Attempt**<br>The action that the role is inspired to perform by the initial event | No indication that the role actually performs an action | Verbs are used in descriptive statements, but these sentences are only related to one initial event (helping the old lady up). | Verbs are used in descriptive sentences, and the sentences are related to the two initial events (helping the elderly woman up and helping to pick up the fallen vegetables). | *Verbs are used in descriptive sentences and the subjects of the two initial events are specific:* the young man and the little boy help the elderly woman, while the office worker (middle-aged person) helps to pick up the fallen vegetables. |
| **Internal response**<br>The role's emotional response to the initial event | No description of emotions. | The feelings are described, but there is no clear connection with the initial event. | The only one feeling is described and there is a clear connection with the initial events. | More than one feeling is described, and there is a clear connection to the initial events. |
| **Consequences**<br>The final outcome of a series of actions related to the initial event performed by the role | No consequence is stated. related to the IEs. | There are statements of consequences, but they are not related to the IEs. | Includes one consequence that is indirectly related to the IEs. | Includes at least one consequence that is directly related to the IEs. |

**Descriptive statistics and bivariate correlations between items**

We used the "comic discourse" paradigm to record participants' narrative texts and scored the macro- and microstructures using quantitative encoding (see Method). The macrostructure was classified into seven items based on the logical development of the story, while the microstructure was classified into ten items according to fundamental language elements. Descriptive statistics of these subcategories as well as the overall dimensions of the synthesized narrative can be found in Supplementary Table 2, and their correlations are presented in Supplementary Table 3. The correlation among different narrative components was relatively low ($r_{mean}$=0.19); however, there was a tendency for greater correlation among items within each structure (macrostructure, $r_{mean}$=0.27; microstructure, $r_{mean}$=0.34).

Supplementary Table 2. Basic statistical information of different subitems for narrative structure

| | N | Mean | SD | Min | Max |
|---|---|---|---|---|---|
| Role | 741 | 1.33 | 0.706 | 0 | 3 |
| Background | 741 | 1.58 | 0.706 | 0 | 3 |
| Initial event | 741 | 1.70 | 0.907 | 0 | 3 |
| Plan | 741 | 0.2240 | 0.78569 | 0 | 3 |
| Attempt | 741 | 1.14 | 0.544 | 0 | 3 |
| Inner reaction | 741 | 0.37 | 0.637 | 0 | 3 |
| Result | 741 | 2.6302 | 0.98549 | 0 | 3 |
| TNC | 741 | 88.14 | 52.236 | 11 | 616 |
| NDC | 741 | 50.83 | 20.950 | 9 | 207 |
| TNW | 741 | 59.71 | 36.487 | 7 | 437 |
| NDW | 741 | 39.25 | 17.816 | 7 | 201 |
| LSL | 741 | 47.89 | 32.878 | 10 | 561 |
| ASL | 741 | 10.03 | 2.03 | 4.20 | 24.00 |
| ACF | 741 | 0.34 | 0.06 | 0.11 | 0.68 |
| AWF | 741 | 0.24 | 0.14 | 0.01 | 1.57 |
| FP | 741 | 1.05 | 1.136 | 0 | 4 |
| GE | 741 | 0.34 | 0.747 | 0 | 4 |

SD = standard deviation; Min = minimum; max = maximum; TNC = total number of characters; NDC = total number of different characters; TNW= total number of words; NDW = total number of different words; LSL = longest sentence length; ASL = average sentence length, ACF = average character frequency; AWF = average word frequency; FP = fluency problems; GE = grammatical error

Supplementary Table 3. Correlation matrix of different subitems for the narrative structure

| | Role | Background | Initial event | Plan | Attempt | Inner reaction | Result | TNC | NDC | TNW | NDW | LSL | ASL | ACF | AWF | FP | GE |
|---|---|---|---|---|---|---|---|---|---|---|---|---|---|---|---|---|---|
| Role | - | | | | | | | | | | | | | | | | |
| Background | .388*** | - | | | | | | | | | | | | | | | |
| Initial event | .378*** | .356*** | - | | | | | | | | | | | | | | |
| Plan | .172*** | .106** | .236*** | - | | | | | | | | | | | | | |
| Attempt | .354*** | .266*** | .494*** | .143*** | - | | | | | | | | | | | | |
| Inner reaction | .260*** | .214*** | .302*** | 0.029 | .318*** | - | | | | | | | | | | | |
| Result | .297*** | .211*** | .306*** | .340*** | .273*** | .133** | - | | | | | | | | | | |
| TNC | .365*** | .224*** | .354*** | .398*** | .364*** | .104** | .510*** | - | | | | | | | | | |
| NDC | .446*** | .318*** | .435*** | .400*** | .402*** | .176*** | .545*** | .949*** | - | | | | | | | | |
| TNW | .340*** | .219*** | .355*** | .400*** | .366*** | .098* | .505*** | .994*** | .941*** | - | | | | | | | |
| NDW | .406*** | .294*** | .419*** | .407*** | .401*** | .141*** | .536*** | .972*** | .984*** | .971*** | - | | | | | | |
| LSL | .301*** | .192*** | .272*** | .379*** | .267*** | 0.073 | .459*** | .918*** | .855*** | .915*** | .891*** | - | | | | | |
| ASL | .306*** | .186*** | .160*** | .087* | .187*** | .125** | .137** | .194*** | .258*** | .169*** | .234*** | .177*** | - | | | | |
| ACF | -.137** | -0.029 | -0.061 | -.146*** | -.163*** | 0.009 | -.203*** | -.289*** | -.293*** | -.277*** | -.280*** | -.252*** | 0.042 | - | | | |
| AWF | -0.075 | 0.075 | -0.042 | -.096* | -.105** | 0.051 | -.122** | -.196*** | -.179*** | -.193*** | -.192*** | -.161*** | 0.057 | .790*** | - | | |
| FP | -.173*** | -.222*** | -.197*** | -.077* | -.135** | -.131** | -0.072 | -0.020 | -0.071 | -0.016 | -0.058 | -0.017 | -.136** | 0.059 | 0.034 | - | |
| GE | -.361*** | -.346*** | -.296*** | -.080* | -.224*** | -.294*** | -.114** | -.081* | -.161*** | -0.069 | -.132** | -0.056 | -.259*** | 0.071 | 0.014 | .453*** | - |

The significant results are noted with asterisks (*** represents p values < .001, ** represents p values < .01, * represents p values < .05, TNC = total number of characters, NDC = total number of different characters, TNW= total number of words, NDW = total number of different words, LSL = longest sentence length, ASL = average sentence length, ACF = average character frequency, AWF = average word frequency, FP = fluency problems, and GE = grammatical error.

Supplementary Table 4. Contributions of various cognitive abilities to different narrative structures

| | (1)<br>Macro | (2)<br>Micro |
|---|---|---|
| Age | -0.098** | 0.060 |
| | (0.010) | (0.009) |
| Education | 0.117*** | 0.133*** |
| | (0.027) | (0.023) |
| Gender | -0.021 | -0.032 |
| | (0.174) | (0.144) |
| General cognition | 0.082* | 0.074 |
| | (0.039) | (0.033) |
| Episodic memory | 0.182*** | 0.078 |
| | (0.010) | (0.009) |
| Spatial construction | 0.134*** | 0.075 |
| | (0.018) | (0.015) |
| Processing speed | -0.056 | -0.058 |
| | (0.003) | (0.003) |
| Executive function | -0.077* | -0.071 |
| | (0.001) | (0.001) |
| Verbal fluency | 0.170*** | 0.205*** |
| | (0.009) | (0.008) |
| *N* | 741 | 741 |

| | | |
|---|---|---|
| $R^2$ | 0.393 | 0.123 |

Note: Controlled for age, sex, education and general cognition. SUR analysis indicated that episodic memory ($\chi^2$ =27.29, p<0.001) and spatial constructional ability ($\chi^2$=10.39, p<0.001) contributed significantly more to macrostructures than to microstructures.

Supplementary Figure 2. SUR analysis results

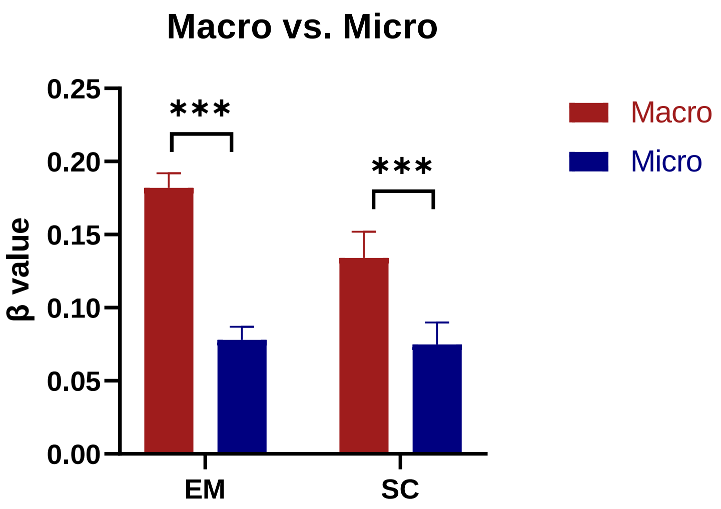


Abbreviation: EM-episodic memory; SC-spatial construction

Supplementary Table 5. Sharpley analysis to rank the cognitive characteristics of different narrative components

| | Macro | | Micro | |
|---|---|---|---|---|
| Factor | Shapley value | Percent | Shapley value | Percent |
| Age | 0.02812 | 7.15% | 0.00278 | 2.25% |
| Education | 0.03407 | 8.66% | 0.03056 | 24.75% |
| Gender | 0.00225 | 0.57% | 0.00019 | 0.16% |
| General cognition | 0.07854 | 19.97% | 0.00921 | 7.46% |
| Spatial construction | 0.05573 | 14.17% | 0.01418 | 11.48% |
| Episodic memory | 0.0354 | 9% | 0.01405 | 11.38% |
| Processing speed | 0.04255 | 10.82% | 0.00587 | 4.75% |

| | | | | |
|---|---|---|---|---|
| Executive function | 0.07223 | 18.36% | 0.00715 | 5.79% |
| Verbal fluency | 0.04044 | 10.28% | 0.03735 | 30.24% |
| Total | 0.393 | 100% | 0.123 | 100% |

Note: The Sharpley analysis was based on the general linear regression in Supplementary Table 4.

Supplementary Figure 3. Nonlinear fitting of the aging trends in different narrative structures

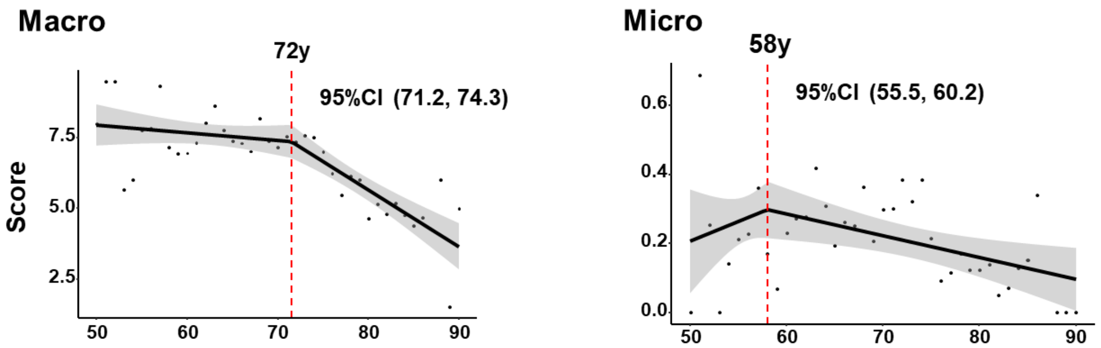


The vertical axis represents scores, with macro scores as the raw scores and micro scores as the standardized scores. The horizontal axis represents age. The red line represents the inflection point.

The adjusted $R^2$ values for these nonlinear models are 0.175 and 0.1072 (macro and micro, respectively), both of which are greater than the explanatory power of the linear model.

Supplementary Table 6. Nonlinear fitting of the aging trends in different narrative structures

| | Macro | | Macro | |
|---|---|---|---|---|
| | LowAge | HighAge | LowAge | HighAge |
| Age | 0.041 | -0.184** | 0.152*** | 0.002 |
| | -0.0168 | -0.0408 | -0.0142 | -0.0324 |
| Education | 0.172*** | 0.086 | 0.163*** | 0.155* |
| | -0.0325 | -0.0526 | -0.0275 | -0.0419 |
| Gender | -0.035 | -0.011 | -0.042 | 0.006 |
| | -0.208 | -0.328 | -0.177 | -0.261 |
| Episodic memory | 0.223*** | 0.099 | 0.075 | 0.048 |
| | -0.0116 | -0.0217 | -0.00983 | -0.0172 |
| Spatial constructional | 0.065 | 0.245*** | 0.076 | 0.062 |
| | -0.0233 | -0.0291 | -0.0197 | -0.0232 |
| Processing speed | -0.02 | -0.11 | -0.055 | -0.035 |
| | -0.00374 | -0.00547 | -0.00317 | -0.00435 |
| Executive function | -0.056 | -0.077 | -0.100* | 0.023 |
| | -0.00151 | -0.0018 | -0.00128 | -0.00143 |
| Verbal fluency | 0.182*** | 0.169* | 0.152** | 0.343*** |
| | -0.0102 | -0.0192 | -0.00865 | -0.0153 |
| General cognition | 0.06 | 0.087 | 0.063 | 0.046 |
| | -0.0487 | -0.0676 | -0.0413 | -0.0538 |
| *N* | 519 | 222 | 519 | 222 |
| $R^2$ | 0.334 | 0.44 | 0.206 | 0.269 |

Macro: the scores of the macrostructure as the dependent variable; Micro: the scores of the microstructure as the dependent variable; N: the number of participants in each group; $R^2$: the proportion of the variability of the dependent variable that can be explained by the independent variable.

Supplementary Figure 4. Age-related trajectories of the narrative and their cognitive contributions

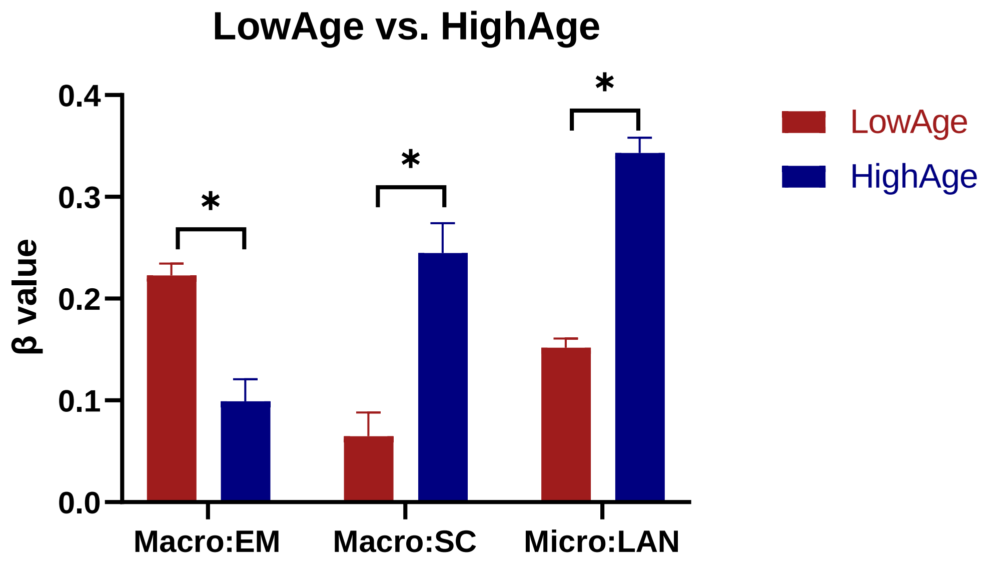


Spatial constructional ability significantly contributes to macrolevel narrative ability ($\chi^2$ =4.37, p<0.05), while the contribution of episodic memory decreases ($\chi^2$ =4.03, p<0.05). Only language skills contribute significantly to microlevel narrative ability with increasing age ($\chi^2$ =5.48, p<0.05)

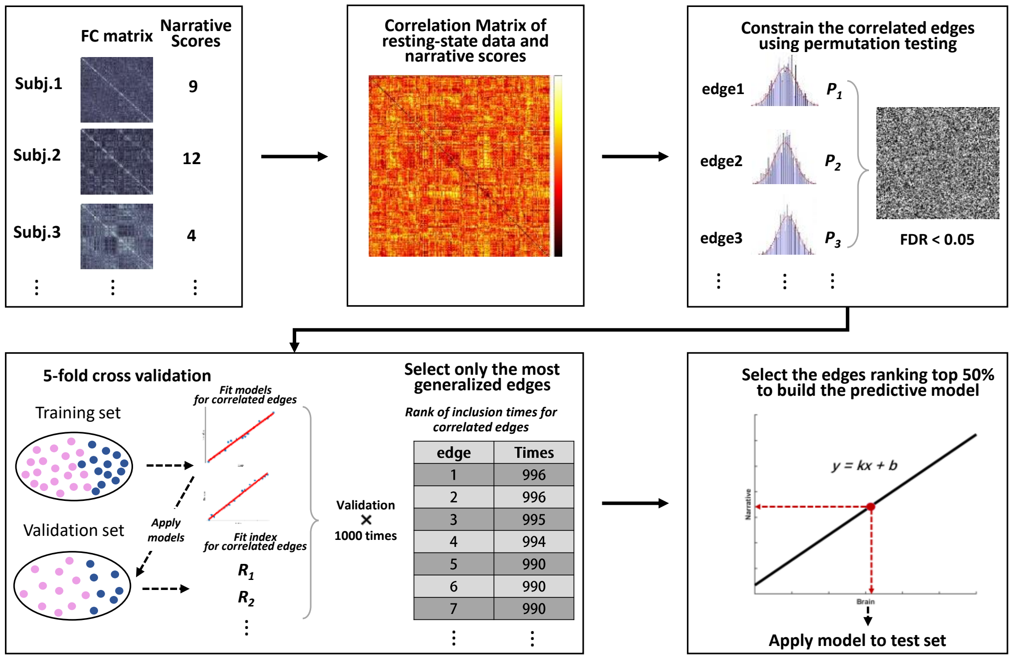


**Supplementary Figure 5.** Initially, the dataset was divided into a training set and a testing set. The inputs for each subject were their resting-state functional connectivity matrix and narrative scores (macro or micro). The correlation matrix was constructed by linear regression of each edge in the connectivity matrices and the narrative measures. After linear regression, the most significant edges were constrained for

further analysis using permutation tests and multiple comparisons correction (false discovery rate, FDR-BH corrected, $P < 0.05$). Next, 5-fold cross-validation was used to select the most generalized edges. The original training set was divided into a new training set and a validation set. The coefficients of each edge predicting narrative measures were fitted across the subjects in the new training set. The edges that satisfied the fitting criteria in the validation set were selected. The process of validation was replicated 1000 times, and the edges most often selected were eventually included. Finally, a predictive model was built that assumes a linear relationship between the validated edges (independent variable) and the narrative (the dependent variable) across the original training set. Next, the corresponding values of the testing set were input into the predictive model. The resulting value was the predicted behavioral measure for the current test subject.

### Using the gradient of human cortical organization to explore the functional hierarchy of different narrative structures

Current evidence suggests the presence of a global gradient in human cortical organization that spans major sensory-motor areas and cross-modal areas[104]. The variation axis of RSFC data in the human brain is captured by a main gradient that extends from primary sensory-motor brain areas to cross-modality brain areas of the DMN. This main RSFC gradient is accompanied by a hierarchy of brain functions ranging from primary sensory processing to higher-order functions. Based on these findings, we conducted a further investigation to determine the specific brain regions within this sensory-DMN gradient that contribute more significantly to macrostructures than to microstructures.

We selected participants with macro- or microstructure scores within the top one-third, termed the HighMacro and HighMicro groups, respectively. We excluded participants who were included in both groups. The final HighMacro and HighMicro groups consisted of 128 and 96 participants, respectively, and there was no significant difference in age between them ($Mean_{macro}$=69.22, $Mean_{micro}$=69.67, t=0.29; $p$=0.77). We then generated the main gradients of these two groups, aligned them to a template

of all participants' gradients, and compared the gradient values of different brain regions between the two groups (see Method).

Supplementary Figure 6. Gradient of human cortical organization exploring the functional hierarchy of different narrative structures

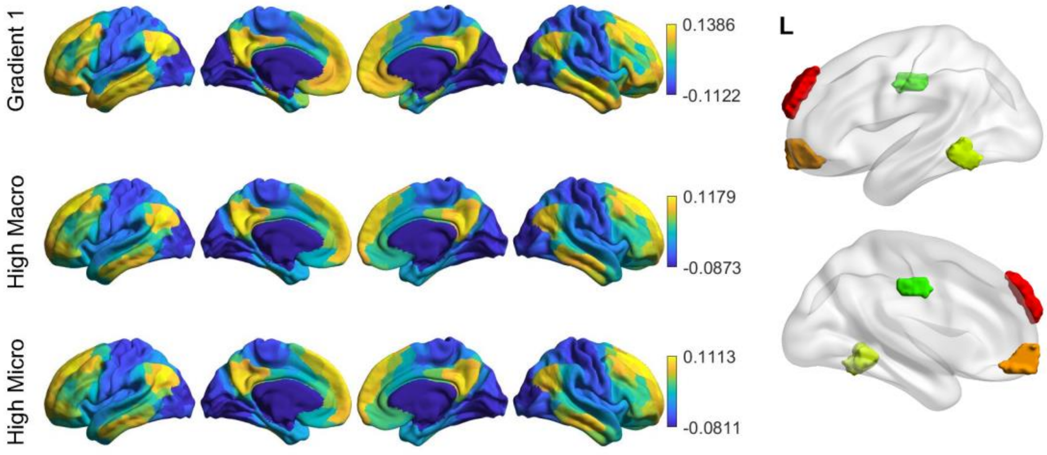


Gradient 1: The human main gradient extends from primary sensory-motor brain areas to cross-modal brain areas of the DMN.

HighMacro: Participants whose macro scores were within the top one third, consisting of 32 participants

HighMicro: Participants whose micro scores were within the top one third, consisting of 24 participants

Supplementary Figure 7. The fitting results of the macro RSFC pattern for predicting different cognitive functions

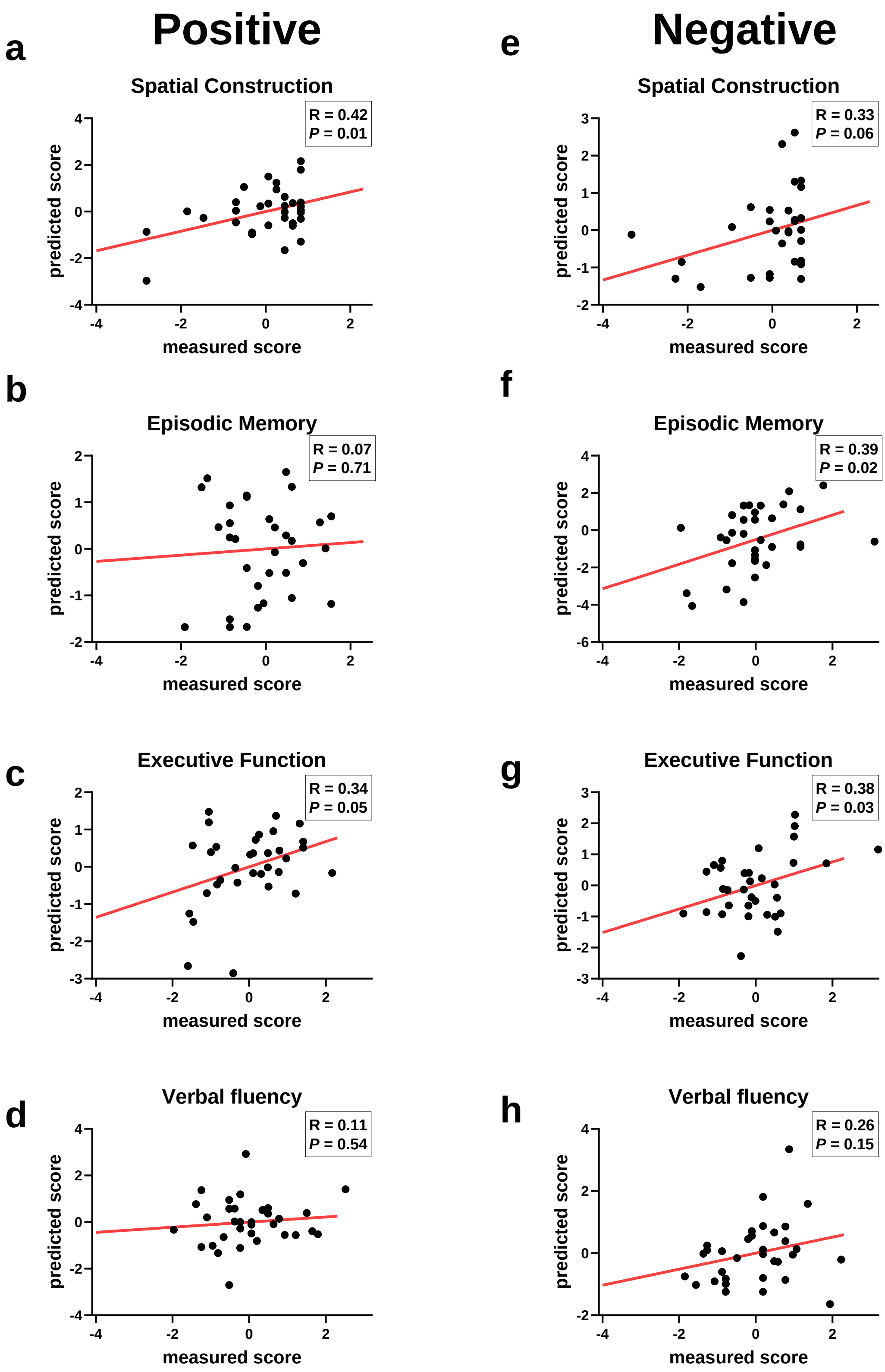


Positive: the regions in Figure 5 exhibiting positive values, indicating that the RSFC pattern is increasingly involved in the narrative process with aging;

Negative: the regions in Figure 5 exhibiting negative values, indicating that the RSFC pattern is decreasingly

involved in the narrative process with aging;

Predicted score: the score predicted for the corresponding cognitive ability using the RSFC pattern in the testing cohort.

Measured score: the actual score corresponding to cognitive ability.

Supplementary Figure 8. The fitting results of the micro RSFC pattern for predicting different cognitive functions

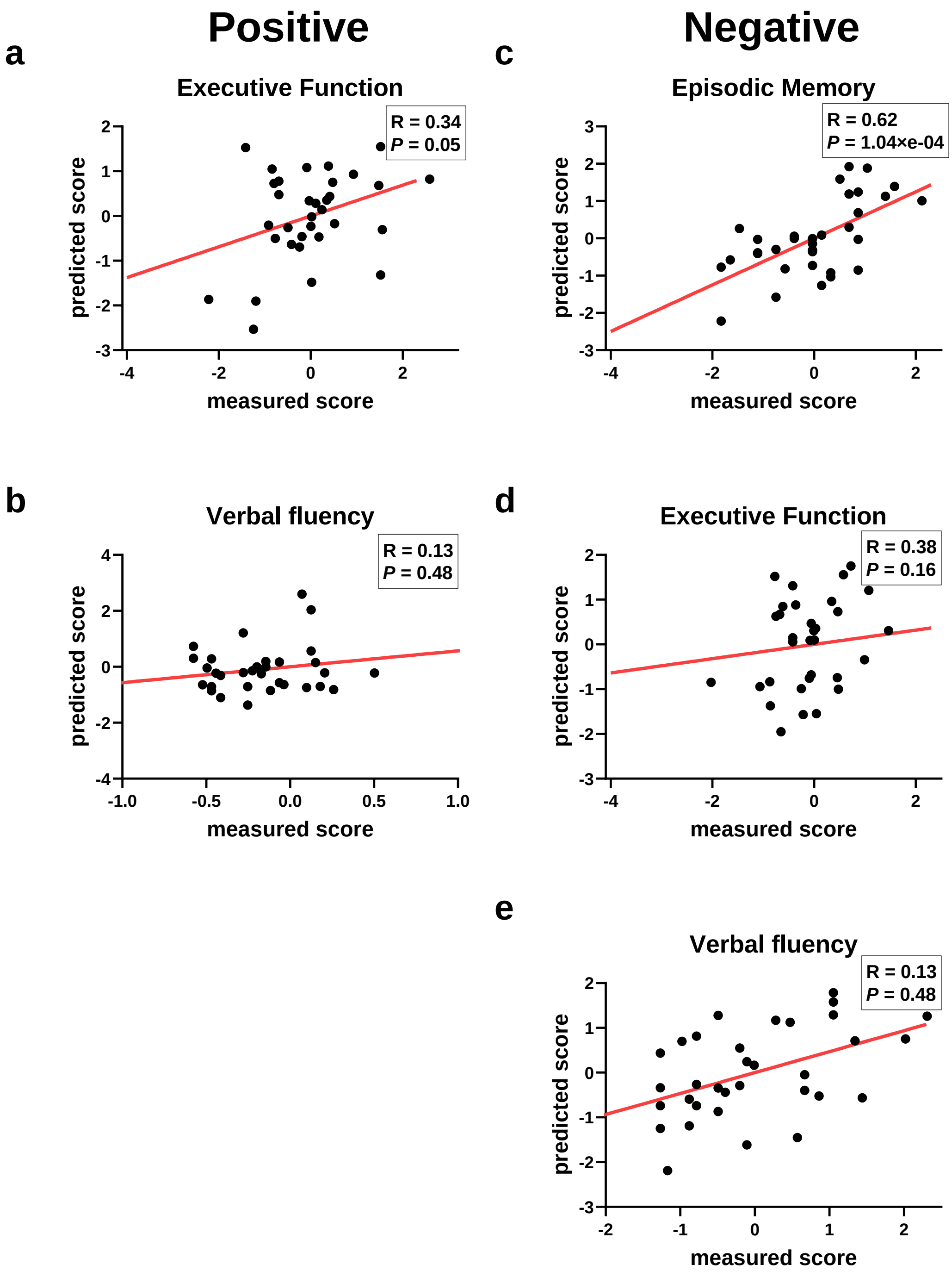


Note: The reason for the absence of positive RSFC predicting VS and memory data in the figure is that the model could not be fitted using linear regression; thus, the fitting index could not be calculated. Similarly, the reason for the absence of a negative prediction of VS is the same. Additionally, one extreme value was removed from the testing

set of the VFT.

**The cognitive contribution of narrative aging**

Additionally, the overlap between hub nodes and negative regions for macrostructure was significant for episodic memory (Dice coefficient=0.37, $P_{permutation}<1\times10^{-04}$), executive function (Dice coefficient=0.15, $P_{permutation}=0.01$) and spatial constructional ability (Dice coefficient=0.20, $P_{permutation}=8.2\times10^{-04}$), while verbal fluency was not significant (Dice coefficient=0.22, $P_{permutation}=0.40$). The hub nodes representing episodic memory had the most significant overlap with negative regions for macrostructure.

In contrast, the overlap between hub nodes and positive regions for microstructure was significant for executive function (Dice coefficient=0.26, $P_{permutation}=5.8\times10^{-04}$) and verbal fluency (Dice coefficient=0.20, $P_{permutation}<1\times10^{-04}$), while episodic memory (Dice coefficient=0.15, $P_{permutation}=0.11$) and spatial constructional ability (Dice coefficient=0.17, $P_{permutation}=0.05$) were not significant. The hub nodes representing verbal fluency had the most significant overlap with positive regions for microstructure. Additionally, the overlap between hub nodes and negative regions for microstructure was significant for episodic memory (Dice coefficient=0.24, $P_{permutation}<1\times10^{-04}$), executive function (Dice coefficient=0.22, $P_{permutation}=6.0\times10^{-04}$), and verbal fluency (Dice coefficient=0.24, $P_{permutation}<1\times10^{-04}$), while spatial constructional ability was not significant (Dice coefficient=0.16, $P_{permutation}=0.06$).

Supplementary Table 7. Gradient of human cortical organization to explore the functional hierarchy of different narrative structures

| | Method 1 | | | | Method 2 | Method 3 | | | | Summary |
|---|---|---|---|---|---|---|---|---|---|---|
| | SC | EM | VFT | EF | Neurosynth | SC | EM | VFT | EF | |
| Macro positive | **R=0.42, P=0.015** | R=0.07, P=0.709 | R=0.11, P=0.54 | R=0.34, P=0.053 | intraparietal sulcus(IPS), posterior parietal cortex, spatial, attention and visual spatial | **DC=0.31, *P*<1.0e$^{-04}$** | DC=0.15, *P*=7.8e$^{-04}$ | DC=0.29, *P*=0.49 | DC=0.28, *P*=8.4e$^{-04}$ | Spatial construction |
| Macro negative | R=0.33, P=0.057 | **R=0.39, P=0.023** | R=0.26, P=0.148 | **R=0.38, P=0.03** | anterior cingulate, dorsal anterior, medial prefrontal lobe, response inhibition, memory | DC=0.20, *P*=8.2e$^{-04}$ | **DC=0.37, *P*<1.0e$^{-04}$** | DC=0.22, *P*=0.40 | DC=0.15, *P*=0.01 | Episodic memory |
| Micro Positive | NaN | NaN | R=0.57, P=5.6e$^{-04}$ | R=0.34, P=0.05 | posterior cingulate, ventral medial, precuneus, default, visual word | DC=0.17, *P*=0.05 | DC=0.15, *P*=0.11 | **DC=0.20, *P*<1.0e$^{-04}$** | DC=0.26, *P*=5.8e$^{-04}$ | Verbal Executive |
| Micro negative | NaN | **R=0.62, P=1.0e$^{-04}$** | **R=0.47, P=0.006** | R=0.37, P=0.160 | inferior frontal, language, phonological, sentences, words | DC=0.16, *P*=0.06 | **DC=0.24, *P*<1.0e$^{-04}$** | **DC=0.24, *P*<1.0e$^{-04}$** | DC=0.22, *P*=6.0e$^{-04}$ | Verbal |

abbreviation: SC-spatial construction; EM-episodic memory; VFT-verbal fluency test; EF-executive function
