# Supplementary material for: Uncovering narrative aging: an underlying neural mechanism compensated through spatial constructional ability
Source: Commun Biol. 2025 Jan 21;8:104. doi: 10.1038/s42003-025-07501-5 (PMC11751312; doi:10.1038/s42003-025-07501-5)
Supplement: Supplementary file 3 — Reporting Summary [file 42003_2025_7501_MOESM3_ESM.pdf]

nature portfolio

Corresponding author(s): COMMSBIO-24-2253

Last updated by author(s): May 21, 2024

Reporting Summary

Nature Portfolio wishes to improve the reproducibility of the work that we publish. This form provides structure for consistency and transparency in reporting. For further information on Nature Portfolio policies, see our Editorial Policies and the Editorial Policy Checklist.

Statistics

For all statistical analyses, confirm that the following items are present in the figure legend, table legend, main text, or Methods section.

n/a	Confirmed	
☐	☒	The exact sample size (*n*) for each experimental group/condition, given as a discrete number and unit of measurement
☐	☒	A statement on whether measurements were taken from distinct samples or whether the same sample was measured repeatedly
☐	☒	The statistical test(s) used AND whether they are one- or two-sided<br>*Only common tests should be described solely by name; describe more complex techniques in the Methods section.*
☐	☒	A description of all covariates tested
☐	☒	A description of any assumptions or corrections, such as tests of normality and adjustment for multiple comparisons
☐	☒	A full description of the statistical parameters including central tendency (e.g. means) or other basic estimates (e.g. regression coefficient) AND variation (e.g. standard deviation) or associated estimates of uncertainty (e.g. confidence intervals)
☐	☒	For null hypothesis testing, the test statistic (e.g. *F*, *t*, *r*) with confidence intervals, effect sizes, degrees of freedom and *P* value noted<br>*Give P values as exact values whenever suitable.*
☒	☐	For Bayesian analysis, information on the choice of priors and Markov chain Monte Carlo settings
☐	☒	For hierarchical and complex designs, identification of the appropriate level for tests and full reporting of outcomes
☐	☒	Estimates of effect sizes (e.g. Cohen's *d*, Pearson's *r*), indicating how they were calculated

Our web collection on statistics for biologists contains articles on many of the points above.

Software and code

Policy information about availability of computer code

Data collection	No commercial, open source and custom code were used to collect data in this study.
Data analysis	The SPM12 (https://www.fil.ion.ucl.ac.uk/spm/software/spm12) and CAT12 (http://www.neuro.uni-jena.de/cat) toolboxes were used to preprocess the anatomical images. The CPM protocol was used to (https://www.nitrc.org/projects/bioimagesuite/) select narrative correlated edges based on a set of single-subject connectivity matrices. The BrainSpace toolbox (https://brainspace.readthedocs.io/en/latest/) was used to identify, visualize, and analyze the large-scale gradients of brain organization among different narrative performance groups. All the text materials were transcribed by an iFlytek machine (https://www.iflyrec.com/zhuanwenzi.html) and then manually proofread by a psychological undergraduate, who is a native Mandarin speaker.

For manuscripts utilizing custom algorithms or software that are central to the research but not yet described in published literature, software must be made available to editors and reviewers. We strongly encourage code deposition in a community repository (e.g. GitHub). See the Nature Portfolio guidelines for submitting code & software for further information.

Data

Policy information about availability of data

All manuscripts must include a data availability statement. This statement should provide the following information, where applicable:

- Accession codes, unique identifiers, or web links for publicly available datasets
- A description of any restrictions on data availability
- For clinical datasets or third party data, please ensure that the statement adheres to our policy

The Neurosynth database is available at https://neurosynth.org/. The raw data expressed in this study could be obtained from the corresponding author. Restriction of raw data is to protect the privacy of participants. Source data are provided with this paper.

Research involving human participants, their data, or biological material

Policy information about studies with human participants or human data. See also policy information about sex, gender (identity/presentation), and sexual orientation and race, ethnicity and racism.

Reporting on sex and gender	The current study describe sex as the biological attribute. All the participants reported their sex.
Reporting on race, ethnicity, or other socially relevant groupings	No socially relevant categorizations were used in the current study.
Population characteristics	Controlled for sex, age and educational levels for the Generalized Linear Model used in the current study.
Recruitment	The participants were recruited during the Beijing Aging Brain Rejuvenation (BABRI) cohort study. The BABRI cohort study is based on the registry of a large community population in Beijing, which collects comprehensive information on aging, tracks changes in cognitive function over years. The subjects volunteered to participate in the narrative task of this study, and finally we recruited more than 740 subjects. All of the participants were 50 years or above at the time of base line enrollment, capable of living independently, without nervous system diseases or psychiatric disorders, having 6 or more years of formal education, which is required for the cognitive assessments.
Ethics oversight	The current study was approved by the Ethics Committees of the State Key Laboratory of Cognitive Neuroscience and Learning in Beijing Normal University (ICBIR_A_0041_002_02).

Note that full information on the approval of the study protocol must also be provided in the manuscript.

Field-specific reporting

Please select the one below that is the best fit for your research. If you are not sure, read the appropriate sections before making your selection.

☒ Life sciences ☐ Behavioural & social sciences ☐ Ecological, evolutionary & environmental sciences

For a reference copy of the document with all sections, see nature.com/documents/nr-reporting-summary-flat.pdf

Life sciences study design

All studies must disclose on these points even when the disclosure is negative.

Sample size	Seven hundred forty-one participants (535 females) aged 50 to 90 years (mean age 68.42±7.87 years) participated in the first behavioral part of the study. The inclusion criteria were as follows: native Mandarin speaker, score ≥24 on the Chinese version of the Mini-Mental State Examination (MMSE), and the ability to complete a battery of neuropsychological tests. Individuals who had a history of major neurological or psychiatric illness were excluded.
Data exclusions	The above participants also underwent fMRI scans. Twenty-three of them were excluded due to excessive head movement (>2mm or >2°). The remaining participants had high-quality resting-state fMRI and T1 MRI data.
Replication	The cross-validation method was used in several places to construct the relationship between the neural substrates and behavioral performance.
Randomization	There was no randomization design in the current study because all the participants were displayed the same materials.
Blinding	Blinding of design was not relevant to the current study because all the participants underwent the same procedure and the MRI scan.

Reporting for specific materials, systems and methods

We require information from authors about some types of materials, experimental systems and methods used in many studies. Here, indicate whether each material, system or method listed is relevant to your study. If you are not sure if a list item applies to your research, read the appropriate section before selecting a response.

Materials & experimental systems

n/a	Involved in the study
☒	☐ Antibodies
☒	☐ Eukaryotic cell lines
☒	☐ Palaeontology and archaeology
☒	☐ Animals and other organisms
☒	☐ Clinical data
☒	☐ Dual use research of concern
☒	☐ Plants

Methods

n/a	Involved in the study
☒	☐ ChIP-seq
☒	☐ Flow cytometry
☐	☒ MRI-based neuroimaging

Plants

Seed stocks

Report on the source of all seed stocks or other plant material used. If applicable, state the seed stock centre and catalogue number. If plant specimens were collected from the field, describe the collection location, date and sampling procedures.

Novel plant genotypes

Describe the methods by which all novel plant genotypes were produced. This includes those generated by transgenic approaches, gene editing, chemical/radiation-based mutagenesis and hybridization. For transgenic lines, describe the transformation method, the number of independent lines analyzed and the generation upon which experiments were performed. For gene-edited lines, describe the editor used, the endogenous sequence targeted for editing, the targeting guide RNA sequence (if applicable) and how the editor was applied.

Authentication

Describe any authentication procedures for each seed stock used or novel genotype generated. Describe any experiments used to assess the effect of a mutation and, where applicable, how potential secondary effects (e.g. second site T-DNA insertions, mosiacism, off-target gene editing) were examined.

Magnetic resonance imaging

Experimental design

Design type

MRI data, including T1-weighted MRI and resting-state functional magnetic resonance (fMRI) scans, were acquired via a Siemens Trio 3T scanner at the Imaging Center for Brain Research at Beijing Normal University.

Design specifications

The resting-state fMRI was designed as the length of 240 TR.

Behavioral performance measures

Narrative
A four-panel comic was used in the study as the narrative material. The comic depicts a story about an old woman who fell down accidentally when she got off the bus and received help from the surrounding people. The instructions required the subjects to familiarize themselves with the pictures first; after the experimenter confirmed that the subjects were ready to tell the story, they were asked to start the recording. The experimenter also asked the participants to confirm the completion of the story and stop the recording. All the text materials were transcribed by an iFlytek machine and then manually proofread by a psychological undergraduate, who is a native Mandarin speaker. The undergraduate student was not aware of the status of the participants. The first author, also a native Mandarin speaker, coded the samples; at the time of coding, the first author was also blinded to the demographic information of the participants.
Cognition
As described in our previous study, all participants underwent a battery of neuropsychological tests at baseline. The assessment involved general cognitive ability and cognitive function across five domains, namely, memory, language, attention, visuospatial abilities, and executive function. General cognitive ability was tested using the Chinese version of the Mini-Mental State Examination (MMSE); memory was tested using the Auditory Verbal Learning Test (AVLT); executive function was tested using the Trail Making Test (TMT); spatial constructional ability was tested using the ROCF-Copy test; and language was tested using the Verbal Fluency Test (VFT).

Acquisition

Imaging type(s): T1-weighted MRI and resting-state functional magnetic resonance (fMRI) scans

Field strength: 3 Tesla

Sequence & imaging parameters: High-resolution T1-weighted, sagittal 3D magnetization-prepared rapid gradient echo sequences were acquired and covered the entire brain (176 sagittal slices, repetition time = 1900 ms, echo time = 3.44 ms, slice thickness = 1 mm, flip angle = 9°, inversion time = 900 ms, field of view = 256 mm × 256 mm, and acquisition matrix = 256 × 256). Resting-fMRI data were acquired using a gradient echo EPI sequence (TE = 30 ms, TR = 2000 ms, flip angle = 90°, 33 slices, slice thickness = 4 mm, in-plane matrix = 64 × 64, field of view = 256 × 256 mm2). The resting scans lasted for approximately 8 minutes, and 240 image volumes were obtained.

Area of acquisition: Whole Brain Scan

Diffusion MRI ☒ Used ☐ Not used

Parameters: Diffusion tensor imaging (DTI) was acquired using a single shot, twice-refocused, diffusion-weighted echo planar imaging sequence coverage of the whole brain, 2 mm slice thickness with no interslice gap, 75 axial slices, TR=8000 ms, TE=60 ms, FOA=60°, acquisition matrix=128×128, 30 diffusion directions with b=1000 s/mm2, and an additional image without diffusion weighting.

Preprocessing

Preprocessing software: For each participant, the first 10 volumes were discarded to allow for adaptation to the magnetic field. Resting data were preprocessed using Statistical Parametric Mapping (SPM; http://www.fil.ion.ucl.ac.uk/spm/)

Normalization: The preprocessing included slice timing, within-subject interscan realignment to correct possible movements, spatial normalization to a standard brain template in the Montreal Neurological Institute coordinate space, resampling to 3 × 3 × 3 mm3, and smoothing with an 8 mm full-width at half-maximum Gaussian kernel. In addition, resting-fMRI data were processed with linear detrending and 0.01-0.08 Hz bandpass filtering and regression correction for nuisance covariates, which included six motion parameters, the global mean signal, the white matter signal, and the cerebrospinal fluid signal.

Normalization template: Montreal Neurological Institute coordinate space

Noise and artifact removal: Friston 24-parameter motion correction (Satterthwaite et al., 2013; Yan et al., 2013), i.e., regression with autoregressive models of motion incorporating 6 head motion parameters, 6 head motion parameters one time point before, and the 12 corresponding squared items (Friston et al., 1996).

Volume censoring: Volume censoring was not performed in this study.

Statistical modeling & inference

Model type and settings: Univariate (linear regression), multivariate (principle component analysis) and predictive (CPM method)

Effect(s) tested: The CPM method(Shen et al., 2017) was used to associate the macro- and micro-structures of narratives with functional connectivity patterns. The PCA algorithm was used to identify the gradients of brain organization among different narrative performance groups to verify the higher functional hierarchy of macrostructures mapped according to RSFC. Fitting the linear regression was used to investigate cognitive function represented by narrative aging-related brain regions.

Specify type of analysis: ☐ Whole brain ☐ ROI-based ☒ Both

Anatomical location(s): To estimate functional connectivity from resting-state fMRI data, we first parcellated the brain into 400 parcels (i.e., nodes) according to the Schaefer 2018 parcellation atlas matched to Yeo 7 networks (Schaefer et al., 2018). Behavioral data suggest a strong correlation between narrative ability and various cognitive processes, particularly episodic memory, executive function, and spatial constructional abilities. Additionally, prior research has demonstrated that the ventromedial prefrontal cortex, orbital frontal cortex, anterior cingulate cortex, and default mode network are the main regions involved in narrative ability. Therefore, we selected the default mode network (DMN), frontoparietal network (FPN), dorsal attention network (DAN) and ventral attention network (VAN) as regions of interest and excluded networks that do not represent high-level cognitive features of narrative ability, such as the visual network87-90. For each participant, the degree of connectivity was estimated by calculating the Pearson correlation coefficient of the 236 nodes' BOLD time series, which resulted in 27730 edges.

Statistic type for inference (See Eklund et al. 2016): Voxel-wise permutation test was used in the current study.

Correction: A permutation test to test for significance (false discovery rate [FDR] BH corrected, $P < 0.05$).

Models & analysis

n/a	Involved in the study
☐	☒ Functional and/or effective connectivity
☐	☒ Graph analysis
☐	☒ Multivariate modeling or predictive analysis

Functional and/or effective connectivity

Pearson correlation and Fisher transformation.

Graph analysis

we performed community detection on the undirected weighted network using the Louvain algorithm. Based on the principle of maximizing modularity, the Louvain algorithm calculates a greedy algorithm to minimize the number of edges within communities and maximize the number of edges between communities.

Multivariate modeling and predictive analysis

We described an algorithm inspired and modified from the CPM protocol37 for selecting narrative correlated edges based on a set of single-subject connectivity matrices, constraining these edges using 5-fold cross-validation of the testing set and finally constructing a predictive model to test the generalization of these edges.
Furthermore, we aimed to provide structural brain validation for the functional neural associations in narrative aging. We extracted the gray matter volume in each node and constructed the structural covariant network of all the subjects (with the hub cluster as the seed point and the nodes associated with narrative aging as the mask). The structural covariant network was correlated with the mean age in each window (successive age windows as above), and the development trend of each edge with increasing age and the structural connectivity of the seed points was obtained. The significant edges after correction were included in the subsequent analysis.
In addition, we selected fiber number as an indicator to construct a white matter network. The edges of the white matter network linked the narrative hub and the nodes involved in narrative aging as the independent variation, while the correlation between these edges in the resting-state functional connectivity network and age was used as the dependent variation. First, we normalized the data and divided it into training and testing sets. Second, the independent variation and dependent variation were calculated in both the training and test sets. We used the correlation between the predicted and actual results to evaluate the model performance. The predicted response variable was calculated on the basis of the test set data by using the regression coefficient and intercepts of the linear model.
